# ASO Author Reflections: Rethinking Local Therapy for Occult Breast Cancer with Advanced Nodal Disease

**DOI:** 10.1245/s10434-026-20299-8

**Published:** 2026-07-29

**Authors:** Courtney M. Lattimore, Helen M. Johnson

**Affiliations:** Department of Breast Surgical Oncology, Division of Surgery, The University of Texas MD Anderson Cancer Center, Houston, TX USA

## Past

Occult breast cancer (OBC) presenting as axillary metastasis without a detectable breast primary is rare, so treatment recommendations have been based on limited data. Historically, mastectomy was used in OBC to remove a presumed breast tumor not identified on mammogram and ultrasound.^1^ Breast magnetic resonance imaging (MRI) now identifies many lesions previously missed by other modalities, leaving a smaller subset of patients with truly MRI-occult disease.^1^ Prior retrospective and population-based studies have suggested that whole-breast radiation may achieve outcomes comparable to those with mastectomy in patients undergoing axillary surgery for OBC, but patients with advanced nodal disease remain underrepresented.^2,3^

## Present

Our study explores whether mastectomy is necessary for patients with OBC and cN2/cN3 disease in the modern era.^4^ In our institutional cohort, nearly all patients underwent MRI and received neoadjuvant systemic therapy.^4^ Most received whole-breast radiation.^4^ Despite infrequent mastectomy, no in-breast or chest wall recurrences occurred.^4^ In a National Cancer Database cohort, overall survival was high among patients treated with whole-breast radiation and in those treated with mastectomy, including those with cN2/cN3 disease.^4^ These findings are consistent with prior studies and meta-analyses supporting breast preservation in selected patients with OBC, showing similar locoregional recurrence, distant metastasis, and mortality with axillary surgery plus radiation versus mastectomy.^3,5,6^

## Future

Given the lack of strong evidence for improved outcomes with mastectomy compared with whole-breast radiation in patients with OBC and advanced nodal disease, future work should define which patients can safely omit mastectomy. Given the rarity of this presentation, multi-institutional registries may be particularly valuable for collecting detailed data on MRI use, biologic subtype, response to neoadjuvant therapy, recurrence, and breast cancer-specific survival, as well as longer follow-up. At this time, patients with OBC should be counseled about the limitations of currently available data, and omission of mastectomy should be considered only for carefully selected patients after multidisciplinary review and shared decision-making.
